# Bio-guided profiling and HPLC-DAD finger printing of *Atriplex lasiantha* Boiss

**DOI:** 10.1186/s12906-018-2416-1

**Published:** 2019-01-03

**Authors:** Tanzeel Zohra, Muhammad Ovais, Ali Talha Khalil, Muhammad Qasim, Muhammad Ayaz, Zabta Khan Shinwari, Sajjad Ahmad, Mohammad Zahoor

**Affiliations:** 1 Department of Biotechnology, Quaid-i-Azam University, Islamabad, 44000 Pakistan; 2 Department of Pharmacy, University of Malakand, Khyber Pakhtunkhwa, 18000 Pakistan; 3 Department of Biotechnology, Pakistan Academy of Sciences, Islamabad, 44000 Pakistan; 4 Department of Eastern Medicine and Surgery, Qarshi University, Lahore, Punjab -56000 Pakistan; 5 Key Laboratory for Biomedical Effects of Nanomaterials and Nanosafety, CAS Center for Excellence in Nanoscience, National Center for Nanoscience and Technology, Beijing, China; 6 Department of Pharmacy, Abasyn University Islamabad Campus, Islamabad, 44000 Pakistan; 7 Department of Chemistry, University of Malakand, Khyber Pakhtunkhwa, 18000 Pakistan; 8 University of Chinese Academy of Sciences, Beijing, 100049 People’s Republic of China; 9 Department of Virology, National Institute of Health, Islamabad, Pakistan

**Keywords:** *Atriplex lasiantha*, Free radicals, Cytotoxicity, Leishmaniasis, Phytochemical analysis

## Abstract

**Background:**

Plants represent an intricate and innovative source for the discovery of novel therapeutic remedies for the management of various ailments. The current study has been aimed to validate the therapeutic potential of ethnomedicinally significant plant *Atriplex lasiantha* Boiss.

**Methods:**

The polarity based extraction process was carried out using fourteen solvents to figure out best extraction solvent and bioactive fractions. Total phenolic-flavonoids contents were quantified colorimetrically and polyphenolics were measured using HPLC-DAD analysis. Moreover, the test samples were tested against several diseases targets following various assays including free radicals scavenging, antibacterial, antifungal, cytotoxic and antileishmanial assay.

**Results:**

Among the solvent fractions, maximum yield was obtained with methanol-water extract i.e., 11 ± 0.49%. Maximum quantity of gallic acid equivalent phenolic content and quercetin equivalent flavonoid content were quantified in methanol-ethyl acetate extract of *A. lasiantha*. Significant quantity of rutin i.e., 0.3 μg/mg was quantified by HPLC analysis. The methanol-ethyl acetate extract of *A. lasiantha* exhibited maximum total antioxidant and total reducing power with 64.8 ± 1.16 AAE/mg extract respectively, while showing 59.8 ± 1.07% free radical scavenging potential. A significant antibacterial potential was exhibited by acetone-distilled water extract of *A. lasiantha* with 11 ± 0.65 mm zone of inhibition against *B. subtilis*. Considerable antifungal activity was exhibited by ethyl acetate-n-hexane extract of aerial part of *A. lasiantha* with 14 ± 1.94 mm zone of inhibition against *A. fumigatus*. Highest percentage of α-amylase inhibition (41.8 ± 1.09%) was observed in ethyl acetate-n-hexane extract. Methanol-acetone extract of *A****.*** *lasiantha* demonstrated significant inhibition of hyphae formation with 11 ± 0.49 mm bald zone of inhibition. Significant in-vitro cytotoxicity against Hep G2 cell line has been exhibited by methanol-chloroforms extract of *A****.*** *lasiantha.*

**Conclusion:**

The current study reveals the prospective potential of *Atriplex lasiantha* Boiss. for the discovery of biologically active compounds through bioassay guided isolation against various diseases.

## Background

Natural resources represent an important reservoir of bioactive compounds for the development of novel drug entities [1–3]. Among natural sources, the plants have been used to cure majority of the ailments since ancient times. The World Health Organization (WHO) has estimated about US $14 billion demand for medicinal plants per year. Approximately 15 to 20% annual growth has also been assessed in the demand of medicinal plants based raw materials, which will increase up to US $5 trillion till 2050 [4, 5]. Various drugs and their derivatives present in current pharmacopoeias have been originated from plants. Twenty eight percent of chemical entities launched during last 20 years were comprised of natural products or their derivatives [6]. Over the globe, number of traditionally used medicinal plants lie between 10,000-53,000; however, few have been evaluated biologically and require further exploration for their complete therapeutic potential [7–9].

Henceforth, nowadays there has been an incredible surge in ethnomedicinal herbal medications [10, 11]. The genus *Atriplex* encompassing about 300 species of angiosperms is well recognized ethnomedicinal genus which exists widely and utilized by aboriginal folks [12, 13]. It belongs to family Chenopodiaceae which grows in Australia, Africa, Asia, and North America. Among these 300 species only eleven species have been reported to be available in Pakistan (Flora of Pakistan). These species are also known as saltbushes, because they are halophytes in nature and have adapted to grow in dry environment with salty soil. Due to their presence in extreme saline condition, a number of species of this group have been researched upon for their pharmacological activities, for example, secondary metabolites isolated from *Atriplex nummularia* and *Atriples leucoclada* possess anti ulcerative, anticolitis, antifungal and mollusicidal activity [14, 15]. *Atriplex farinose* possesses antifertility potential, it also has anti ulcerative and anticolitis activity [16]. *Atriplex conferitifolia* is said to possess a noteworthy activity against cervical cancer cells of human and have analogous potential to a cancer drug called “Onxol” which is FDA approved [17]. It has been found that the alkaloids that are extracted from *Atriplex vesicaria* show antileukemic activity [18], whereas saponins (triterpenoids) extracted from *Atriplex glauca* exhibit cytotoxicity against human colon cancer cells [19]. *Atriplex semibaccata, Atriplex portulacoides, Atriplex parvifolia and Atriplex inflate* have been reported to have insecticidal activity against Tribolium spp. [20]. Leaves of *Atriplex canescens* are used to cure infections of gastrointestinal in Mexico and its methanolic extract show anti-bacterial activity against *E.coli* and *Salmonella typhimurium* [21]. In Pakistan, the leaves of *Atriplex crossifolia* are used in the treatment of yellow jaundice and throat infection [22].

Presence of diverse secondary metabolites and their medicinal importance motivated us to explore the species *Atriplex lasiantha* of genus *Atriplex. A. lasiantha* Boiss. (Orache) is an annual erect branched herb, having deep spreading roots. It is distributed in wet mountains, deserts, arid semi-arid mountains. It is also found in the plains of Indus and Balochistan and is mainly used for food and forage. The current study compiles an exclusive report on antioxidant, antibacterial, antifungal, antidiabetic, antileishmanial and cytotoxic activity of *Atriplex lasiantha* Boiss. by using multimode polarity extraction system.

## Methods

### Chemicals and reagents

In this study, solvents that were used for extraction purpose were of analytical grade. The solvents included dimethyl sulfoxide, methanol, acetone, ethyl acetate, ethanol, chloroform, and n-hexane. The solvents and reagents like dipotassium hydrogen phosphate, potassium dihydrogen phosphate, ferric chloride, trichloroacetic acid, ammonium molybdate, potassium ferricyanide, sulphuric acid, aluminium chloride and potassium acetate were purchased from Merck, Darmstadt, Germany. 2,2-diphenyl-1-picryhydrazyl (DPPH) and Folin-Ciocalteu reagent were bought from Sigma–Aldrich (Steinheim, Germany). Tween-20 was bought from Merck (Schuchardt, USA), while Phosphate Buffer Saline (PBS), nutrient agar, Sabouraud dextrose agar (SDA), Ttryptone Soy Broth (TSB),sterile normal saline solution (0.9%), sea salt, 0.5% triton x-100, roxithromycin and cefixime, clotrimazole and amphotericin B, doxorubicin, vincristine, surfactin, were purchased from Sigma (Sigma Aldrich, USA), Medium ISP4 (was Prepared in lab), and those reagents that were used i.e.myricetin, gallic acid, rutin, kaempferol catechin,caffeic acid, quercetin, ascorbic acid, were also purchased from Merck, Darmstadt, Germany.

### Cultures and cell line

The bacterial strains used in biological assays were Gram positive strains: *Staphylococcus aureus* (ATCC 6538), *Micrococcus luteus* (ATCC 10240) and *Bacillus thuringiensis (*ATCC 6633) and Gram negative strains: *Klebsiella pneumonia* (ATCC 1705), and *Escherichia coli (*ATCC 25922). Whereas,fungal strains that were used as test organism were *Fusarium solani (*FCBP 0291), *Aspergillus flavus* (FCBP 0064), *Aspergillus fumigates* (FCBP 66), *Mucor* species (FCBP 0300) and *Aspergillus niger* (FCBP 0198). HepG2 cancer cell line (RBRC-RCB1648), *Leishmania tropica* (kMH 23 strain) and *Streptomyces* (85Estrain) were also used.

### Collection and identification of plant

The sample of *A. lasiantha* was collected from Peshawar, Khyber Pakhtunkhwa (KP) Pakistan and identified and verified by Prof. Dr. Mushtaq Ahmad, Department of Plant sciences, Faculty of Biological Sciences, Quaid-i-Azam University Islamabad. Plant was selected on the basis of its ethnopharmacological significance, safety profile in humans, cost effectiveness, ease of availability and was deposited in MoSAEL Lab’s herbarium, Quaid-i-Azam University Islamabad, Pakistan (voucher number MOSEAL-347). Aerial part of *A. lasiantha* was apportioned from any deteriorated or foreign matter. Afterwards, they were dried in well-ventilated area at room temperature for four weeks and coarse grinded via cutter machine [23, 24].

### Extract preparation

Initially, the plant material was washed with water to remove dirt particles and then shade dried for four weeks at room temperature. After that aerial part was grinded by using electric knife mill and then stored in tight jar. The powered plant material was subjected to extraction followed by sonication and maceration with analytical grade solvents i.e., N: n-hexane, C: Chloroform, EA: Ethyl acetate, A: Acetone, E: Ethanol, ME: Methanol, W: Distilled water, NEA: *n*-hexane: ethyl acetate, EN: ethanol: *n*-hexane, MEC: methanol: chloroform MEEA: Methanol-Ethyl acetate, MEA: methanol: acetone, AW: acetone: water, MED: methanol: water. Fourteen different solvents of wide ranging polarity were used, includes seven single solvent and seven in combinations (1:1). Plant powder (30 g) was socked in 120 mL of respective solvents for maceration in separate Erlenmeyer flasks and left for 3 days at room temperature. Shaking was done on daily basis followed by ultrasonication at a frequency of 40 KHz. After 3 days, filtration was done by Whatman No.1 filter paper and next same volume was added. Second volume of respective solvents was filtered after two days and third volume of solvent was added. Third volume of solvent was again filtered after two days, and all filtrate was placed at room temperature for drying. The filtrates were vaporized accordingly and the dried plant extracts were then stored in pre-weighed labeled vials at − 20 °C for further analysis [25].

### Percent extract recovery

The percentage yield of dried crude extracts recovery after extraction was evaluated gravimetrically by using this formula which are as follows.

$$ \mathrm{Percent}\kern0.17em \mathrm{extractrecovery}=\frac{\alpha }{2}\times 100 $$

Where “a” and “b” corresponds to the weight of dried extract and weight of plant powder used respectively.

### Quantitative phytochemical screening

#### Total phenolic content (TPC)

Total Phenolic Content present in plant sample was calculated by using already reported spectrophotometric method [26]. Briefly, 20 μL of plant samples were added to 90 μL Folin Ciocalteu reagent in 96 well plate. The resultant mixture was incubated for 5 min followed by addition of 90 μL of Na_2_CO_3_. Subsequently, the reaction mixture was incubated for 30 min at 37 °C. The absorbance of reaction mixture was recorded at 630 nm with the help of microplate reader (Biotech USA, microplate reader Elx 800). The standardization curve (y = 0.0136x + 0.0845, R^2^ = 0.9861) of gallic acid was used for quantification of phenolic content. Resulting phenolic content was expressed in term of gallic acid equivalent (μg GAE/mg extract).

#### Total flavonoid content (TFC)

Total Flavonoid Content present in plant sample was calculated by using already reported method which was based on formation of aluminum chloride complex [27]. Test sample (20 μL) was added followed by 10 μL AlCl_3,_ 10 μL of 1 M KCH_3_CO_2_ and 160 μL of H_2_O in 96 well plate. Incubated for about 30 min after that absorbance of reaction mixture was recorded at 415 nm with the help of microplate reader (Biotech USA, Elx 800 microplate reader). The standardization curve (y = 0.0268x + 0.00764, R^2^ = 0.9986) of quercetin was used for quantification of flavonoid content. Resulting flavonoid content was expressed in term of quercetin equivalent (μg QE/mg extract).

#### Quantitative analysis via HPLC-DAD

For interpretation of significant manifestation of phenolics and good biological activity, we selected MEEA extract for HPLC-DAD analysis. Chromatographic analysis of *A. lasiantha* extract for polyphenols were carried out by following already reported method [28]. In this High-performance liquid chromatography diode-array detection system, (DAD; Agilent technologies, Germany) was equipped with Agilent chem station Rev. B.02–01-SR1 (260) and Zorbex C8 analytical column (4.6 × 250 mm, 5 μm particle size. Agilent, USA). Plant samples were prepared in 10 mgmL^−1^methanol and standards i.e.Gallic acid, rutin, quercetin, catechin, caffeic acid, apigenin, myricetin and kaempferol were prepared in methanol and then diluted to get final concentration of 10, 20, 50, 100, 200 μg/ml respectively with the help of sonication in ultrasonic water bath. The gradients system utilized was comprised of solvent A (methanol: acetic acid; deionized water, 100: 20: 180, *v*/v) and solvent B (methanol: acetic acid: deionized water, 900: 20: 80,v/v). The flow rate was kept at 1 mL/min. Each sample solution (20 μL) was loaded into column. Before the injection, each sample was filtered with 0.2 μm membrane filter (sartolon polyamide). The gradient volume of B was utilized as follows: 0–50% in 0–20 min, 50–100% in 20–25 min and then 100% from 25 to 30 min. The absorption of samples was recorded at different wavelengths e.g., gallic acid (257 nm) catechin (279 nm), rutin (257 nm), caffeic acid (325 nm) and kaempferol, quercetin, myricetin (368 nm). The reconditioning of column was done for 10 min before each analysis. By using the standard, integration of peak has been done for quantification of polyphenols. All chromatography was processed at an ambient temperature.

### Antioxidant assays

#### Percentage radical scavenging activity

Radical scavenging activity of *A. lasiantha* extracts were estimated by measuring the change in 2, 2-diphenyl-1-picryhydrazyl (purple colored) to diphenyl picrylhydrazine (Yellow Colored). The spectrophotometric method was used for investigation [29, 30]. As reference standard, ascorbic acid was used. DPPH at a concentration of 180 μl (9.2 mg/100 ml methanol) was mixed with 20 μL of test sample and incubated for one hour in dark at 37 °C. The absorbance of reaction mixture was recorded at 517 nm with the help of microplate reader (Biotech USA, microplate reader Elx 800) and was estimated by using formula that was as:

$$ \%\mathrm{scavengingactivity}=1\hbox{-} \frac{\mathrm{AbS}}{\mathrm{AbC}}\times 100 $$

Where the “Ab_S_^”^and “Ab_C_”correspond to absorbance of sample and control respectively.

#### Total antioxidant capacity

Total antioxidant capacity was estimated by phosphomolybdenum method [31]. Reagent was composed by mixing 0.6 M H_2_SO_4_, 4 mM (NH_4_)_6_ MO_7_O_24_.4H_2_O and 28 mM NaPO_4._100 μL aliquot of each sample was mixed with 1 mL solution of the reagent. After that the reaction mixture was incubated at 90 °C for 95 min. Ascorbic acid and DMSO were used as positive control and as negative control respectively The absorbance was recorded at 645 nm with the aid of microplate reader (Biotech USA, microplate reader Elx 800) and was expressed in term of ascorbic acid equivalent per microgram extract (μg AAE/mg extract).

#### Total reducing power

The reducing capacity of plant extracts was assessed by formerlydefined method [32] with small modification. Extracts samples (20 μL) were mixed with 0.5 mL of PBS buffer and 1% potassium ferricyanide followed by incubation for 20 min at 50 °C. Afterward, 0.5 mL of C_2_HCl_3_O_2_ was added and resultant mixture was centrifuged at 3000 rpm for 10 min. Lastly, the supernatant was mixed with 0.5 mL FeCl_3_ followed by 0.1 mL distilled water. The absorbance of reaction concoction was recorded at 700 nm with the aid of microplate reader (Biotech USA, Elx 800 microplate reader) and was expressed in term of ascorbic acid equivalent per microgram extract (μg AAE/mg extract).

### Antimicrobial activity

#### Antibacterial assay

Disc diffusion technique was used for investigation of bacterial susceptibility of plant extract as previously reported [33, 34]. Five strain were used i.e. Gram positive strains: *S. aureus* (ATCC 6538), *Micrococcus luteus* (ATCC 10240) and *Bacillus thuringiensis (*ATCC 6633) and Gram negative strains: *Klebsiella pneumonia* (ATCC 1705), and *Escherichia coli (*ATCC 25922). Sterile loopful full of spores was inoculated in 10 mL aliquot of nutrient broth followed by incubation at 37 °C for 24 h. After that, turbidity of each inoculum was maintained at 10^4^ CFU/mL by comparing it with 0.5 McFarland turbidity BaSO_4_ standards. A volume of microbial preparation (100 μL) was spread on agar plates with subsequent addition of 100 μg of sample to the plates and incubated for 24 h at 37 °C. After this clear zone of inhibition around the discs was recorded with vernier caliper to the nearest. Cefixime and DMSO were used as positive and negative control respectively.

#### Antifungal assay

Disc diffusion method was used with for investigation for the fungus susceptibility of plant extract [35, 36]. Five strains were used i.e. *Fusarium solani* (FCBP 0291), *Aspergillus flavus* (FCBP 0064), *Aspergillus fumigatus* (FCBP #66), *Mucor* species (FCBP #0300) and *Aspergillus niger* (FCBP 0198). Initially fungal spores were refreshed by swabbing on sterile SDA plates followed by incubation for 5–7 days at 28 °C. After this, spores of fungal strains were harvested in 0.02% Tween-20 solution and their turbidity was maintained at 10_4_ CFU/mL by comparing it with 0.5 McFarland turbidity BaSO_4_ standards. A suspension (100 μL) of test strain was spread on sterile SDA plate then 100 μg/ml of sample was poured on seeded plates followed by incubation for 28 °C for 24–48 h. After this clear zone of inhibition around the discs was recorded with Vernier caliper to the nearest. Clotrimazole and DMSO were used as a positive and negative control respectively.

#### Broth micro-dilution method

Selected samples (≥10 mm zone of inhibition) were further evaluated for minimum concentrations (MIC) by using standardized 3- fold micro dilution method. Bacterial suspension (50 ml) of each tested bacteria were grown in broth and then added in 96 well plate with different concentrations if the test samples (100 μg/ml to 7.40 μg/ml). The final volume in the well was kept 200 μL. After the incubation period, plates were analyzed by ELISA Spectrophotometer to determine the growth of bacteria in each respected well. The minimum concentration of each sample that was showing no growth are taken as MIC (Minimum inhibitory concentration).

#### Alpha amylase inhibition assay

For determination of amylase inhibition spectrophotometric method was used with that was previously described [37]. Phosphate buffer (15 μL), 25 μL of α-amylase enzyme, 10 μL samples (100, 50, 25, 12.5 and 6.25 μg/ml concentrations respectively) and 40 μL starch was added in successive steps monitored by incubation for 30 min at 50 °C. After incubation, lastly 20 μL of 1 M hydrochloric acid and 90 μL of iodine solution were added. Acarbose and DMSO were castoff as positive and negative control respectively. The absorbance of reaction mixture was recorded at 540 nm with the help of microplate reader (Biotech USA, Elx 800 microplate reader). Percentage enzyme inhibition was measured by using this formula and results was expressed in term of % enzyme inhibition per mg of plant extract.

$$ \mathrm{Enzymeinhibition}=\frac{\mathrm{AbS}\hbox{-} \mathrm{AbN}}{\mathrm{AbB}\hbox{-} \mathrm{AbN}}\times 100 $$

Whereas, “Ab_S_”_,_ “Ab_N_”and “Ab_B_”corresponds to absorbance of sample, negative control and blank respectively.

#### Hemolytic activity

Hemolytic activity was carried out by using fresh isolated blood from human body. For isolation of RBCs, 1 mL fresh blood was centrifuged at 14, 000 rpm for 5 min. After that, 200 μL from it was mixed up with PBS (pH = 7.2) for preparation of erythrocyte suspension. Test sample (100 μL) was added in eppendorf containing 100 μL erythrocytes suspension followed by incubation for 60 min at 35 °C. After incubation, the reaction mixture was again centrifuged at 10,000 rpm/10 min.The absorbance was recorded at 530 nm with the help of microplate reader (Biotech USA, Elx 800 microplate reader) to find out the percent hemoglobin release. Triton X-100 and DMSO were used as positive and negative control respectively. All experiments were repeated three times and results were expressed in term of % Hemolysis per mg of plant extract. Hemolysis was find out by using the following formula:

$$ \%\mathrm{Hemolysis}=\frac{\mathrm{AbS}\hbox{-} \mathrm{AbNC}}{\mathrm{AbPC}\hbox{-} \mathrm{AbNC}}\times 100 $$

Whereas, “Ab_S_”_,_ “Ab_NC_”and “Ab_PC_”corresponds to absorbance of sample, negative control and positive control respectively.

#### Protein kinase inhibition assay

*Streptomyces* (85E strain) cultured in minimal ISP4 media was used for this assay [38]. Test sample (100, 50, 25, 12.5 and 6.25 μg/ml) was added to freshly seeded plates followed by incubation for 72 h at 30 °C. After incubation, zone of growth inhibition was measured. The development of bald zone around the disc suggested inhibition of hyphae formation while clear zone shows cytotoxicity by killing *Streptomyces*. Surfactin and DMSO was used as positive and negative control respectively.

#### Anticancer activity

The in-vitro cytotoxic activity against Hep G2 cancer cell line (RBRC-RCB1648) was evaluated with the help of standard protocol [24, 39]. Test sample (20 μL of 100, 50, 25, 12.5 and 6.25 μg/ml concentration) and PBSA (20 μg/mL) were added to 180 μL culture followed by incubation for 72 h at 37 °C in a CO_2_ humified incubator. The reaction was stopped with the addition of 50 μL of cold 20% TCA and then again incubated for 60 min at 4 °C for cell fixation. Subsequently, the cells become immobilized, washed with tap water four times followed by staining with 50 μL of 0.057% SRB in 1% acetic acid at room temperature. Wells were again placed overnight for drying and washed four times with 1% acetic acid. Subsequently, 0.2 mL test sample of 10 mM Tris base (pH 10) was then added and left for one hour to solubilise the bounded dye. Doxorubicin and DMSO were used as positive and negative control respectively. In order to evaluate the percentage of cells survival, microplate reader (Biotech USA, Elx 800 microplate reader) was used at 515 nm for measuring the optical density. Percentage of cell growth inhibition was determined by using formula:

$$ \%\mathrm{inhibition}=100-\left[\left(\mathrm{ODcells}+\mathrm{samples}\hbox{-} \mathrm{ODday}\kern0.24em 0\right)/\left(\mathrm{ODcells}+\mathrm{DMSO}\kern0.17em \mathrm{ODday}0\right)\times 100\right] $$

Where “OD” corresponds to optical density. 0 day control was conceded by adding same number of cells followed by incubation at 37 °C for 1 h and followed the same protocol.

#### Antileishmanial assay

MTT colorimetric assay as described previously [40] was used by using *Leishmania tropica* (KMH 23 strain) culture supplemented with bovine fetal serum. Density of culture was maintained at 1 × 10^6^ promastigotes per mL. Test sample (20 μL) at final concentration of 100 μg/ml was added to 180 μL of culture followed by incubation at 24 °C for 72 h in 5% CO_2_ humified incubator. The absorbance was recorded at 540 nm with the assistance of microplate reader (Biotech USA, Elx 800 microplate reader). The lived promastigotes were counted under inverted microscope with the help of hemocytometer and their LC_50_ was calculated by table curve (2D ver. 4 software). DMSO and Amphotericin B were used as negative and positive control respectively. Percentage mortality was calculated by using the formula:

$$ \%\mathrm{mortality}=1-\frac{\mathrm{AbS}}{\mathrm{AbC}}\times 100 $$

Where “Ab_S_” and “Ab_C_”corresponds to absorbance of sample and absorbance of control respectively.

### Statistical analysis

Multiple group comparison was performed by Two way ANOVA followed by Bonferroni post test by GraphPad Prism Version 5.0 (Harvey Motulsky President, GraphPad Software Inc.) in which the *P* < 0.05 were considered significant. The data was analyzed via Statistix 8.1 software, expressed as mean ± standard deviation. Correlation investigation was conceded out by using regression line in Microsoft Excel 2013 Program. IC_50_ and LC_50_ were calculated by table curve 2D version 4 software. Graphs were plotted by Origin 8.5 software.

## Results

### Effect of solvent on extract yield

A total of 14 different extracts from the aerial parts of *A. lasiantha* were prepared in different solvents and investigated to explore the spectrum of phytochemicals and biological activities. Percentage recovery for different solvent extracts is summarized in Table 1. Maximum amount of extract was recovered from methanol-water combination with an extract yield of 11% (*w*/w) followed by 10 and 7.7% for methanol-ethyl acetate and methanol extracts respectively. Whereas, n-hexane fraction yielded the least content (1.7%) because of being least polar among the solvents used.

**Table 1:** Percent extract recovery of *A. lasiantha* extracts through different extraction solvents

E. Sol	N	C	EA	A	E	M	W	NEA	EN	MEC	MEEA	MEA	AW	MD
% E. Rec.	1.7± 1.08	6.8± 2.03	1.9± 1.01	4.9± 1.48	2.9± 1.71	7.7± 0.90	6.1± 0.95	5.6± 0.43	3.7± 1.03	5.3± 0.78	10± 1.03	3.3± 1.09	4.96± 0.73	11± 0.49

Results were presented as mean ± standard deviation through triplicate experiments. N: *n*-hexane, C: Chloroform, EA: Ethyl acetate, A: Acetone, E: Ethanol, ME: Methanol, W: Distilled water, NEA: *n*-hexane: ethyl acetate, EN: ethanol: *n-* hexane, MEC: methanol: chloroform MEEA: Methanol-Ethyl acetate, MEA: methanol: acetone, AW: acetone: water, MD: methanol: water

### Quantitative phytochemical screening

#### Total phenolic and flavonoid content

**Fig. 1:**
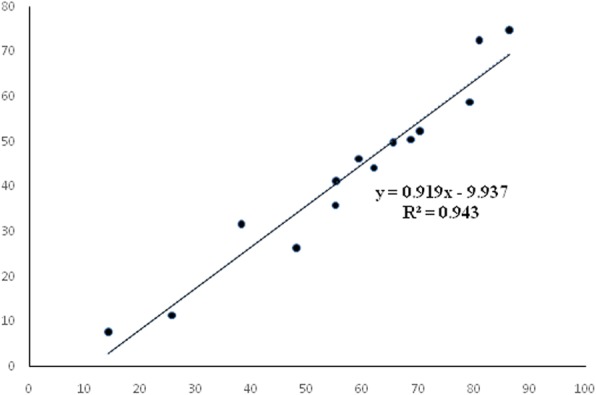
Graph representing correlation of Total Phenolic Content and Total Flavonoid Content in terms of (μg GAE/mg) and (μg QE/mg) respectively

**Fig. 2:**
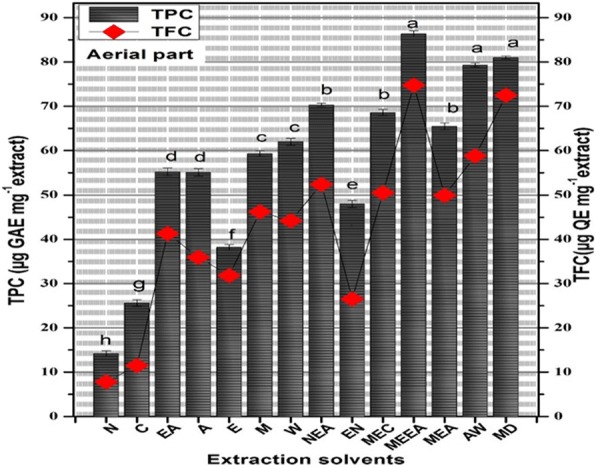
Total phenolic content and flavonoid content determination of aerial part of *A. lasiantha.* Results were presented as mean ± S.D. (*n* = 3). N: n-hexane, C: Chloroform, EA: Ethyl acetate, A: Acetone, E: Ethanol, ME: Methanol, W: Distilled water, NEA: *n*-hexane:ethyl acetate, EN: ethanol:*n*-hexane, MEC: methanol:chloroform MEEA: Methanol-Ethyl acetate, MEA: methanol:acetone, AW: acetone:water, MED: methanol: water

The profile of total phenolic and flavonoid content was determined from standard curve calibration of gallic acid and quercetin respectively. The TPC and TFC has been provided in Fig. 1 while their correlation has been depicted in Fig. 2. The correlation coefficient value of 0.943 demonstrates the comparable quantity of TPC and TFC in all the test samples. The phenolic and flavonoid chemical entities present widespread in plant kingdom with potential antioxidant nature. ROS like super oxides, hydroxyl radicals, hydrogen peroxides can be frequently generated inside the body having the tendency to harm proteins, lipids etc., in the body. The antioxidants induces their therapeutic effect by neutralizing the ROS species and therefore TPC and TFC were carried out. Highest content of gallic acid equivalent phenolic entities i.e., 86.4 ± 1.41 μg GAE/mg extract and 81 ± 1.31 μg GAE/mg extract were observed in methanol-ethyl acetate extract and in methanol-water extracts respectively. The phenolic content decreased because of extraction solvent polarity in accordance with the following order followed by MEEA > MEW > AW > EAN > MEC > MEA > W > ME > EA > A > E > EN > C > N. Whereas, highest flavonoid content 74.8 ± 0.654 μg QE/mg extract was observed in the methanol-ethyl acetate extract of aerial part of *A. lasiantha* followed by MW > AW > EAN > MEC > MEA > W > ME > EA > A > E > EN > C > N.

#### Quantitative analysis via HPLC-DAD

**Fig. 3:**
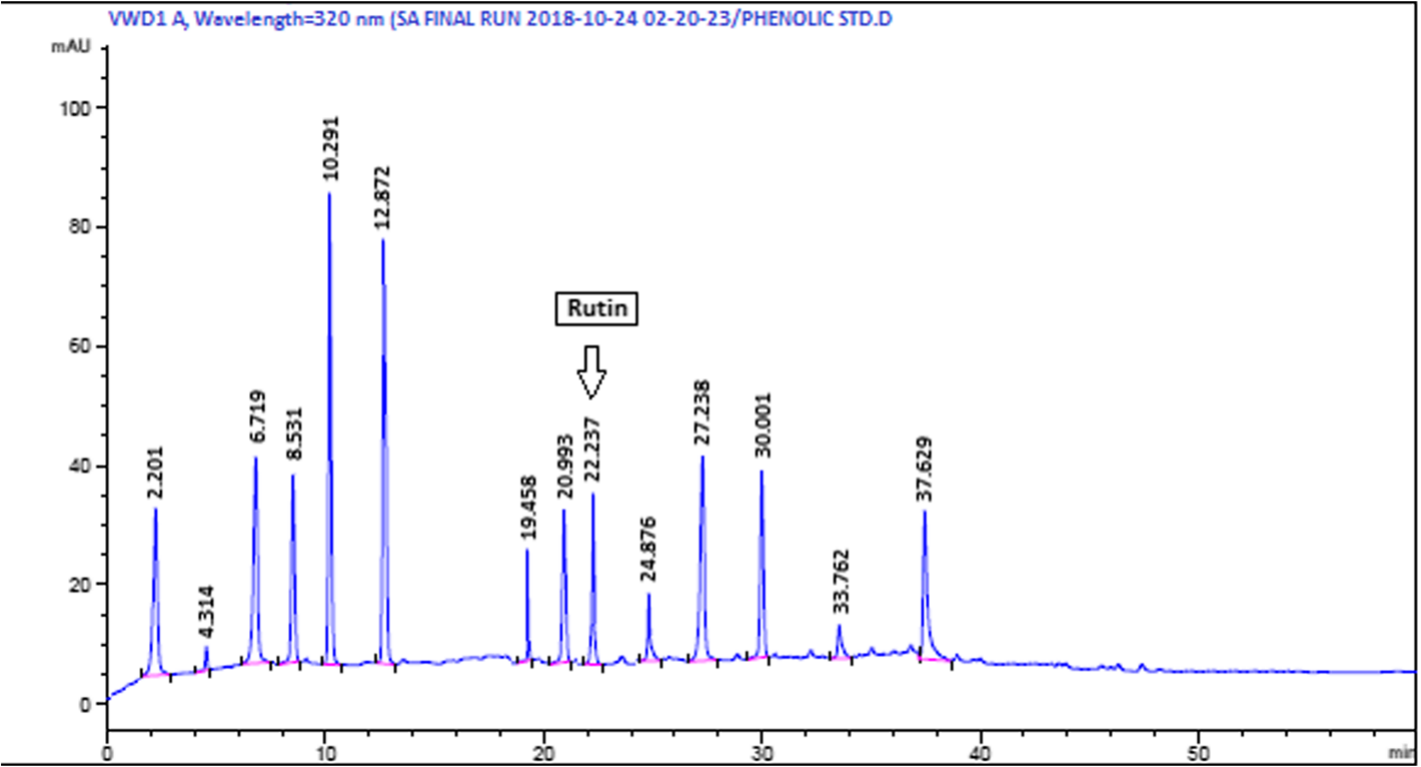
HPLC- DAD profiling of *Atriplex lasiantha* methanol: ethyl acetate extract (AMEA) at different wavelength. Only rutin was detected at 257 nm. Conditions: Mobile Phase A (methanol: acetic acid; deionized water, 100: 20: 180, *v*/v) and Mobile Phase B (methanol: acetic acid: deionized water, 900: 20: 80,v/v). The flow rate was kept at 1 ml/min. The gradient volume of B was utilized as follows: 0–50% in 0–20 min, 50–100% in 20–25 min and then 100% from 25 to 30 min. The absorption of samples was recorded at different wavelengths e.g. Gallic acid at 257 nm, catechin at 279 nm, Rutin at 257 nm, caffeic acid at 325 nm and kaempferol, quercetin, myricetin at 368 nm, but only rutin was detected

Fingerprinting the chemical profile of analytes via HPLC is considered as an unpretentious, reproducible, profound and reliable method [32]. Considering the vacuum of knowledge and significant TPC and TFC results, it was important to study the polyphenols in detail. Polyphenols have well established antioxidant properties and can interfere with the regulation of redox enzymes. The crude extracts were analyzed quantitatively by reverse phase HPLC technique for the identification and quantification of polyphenols (Fig. 3). The peaks of samples were compared with the retention time and UV absorption spectra of reference compounds including polyphenols i.e. caffeic acid, apigenin, gallic acid, myricetin and kaempferol. On account of noteworthy presence of phenolics and good biological activity, we selected MEEA extract for HPLC analysis. The flavonoids that are under analysis possess noteworthy biological activities but from analysis only rutin (0.3 μg/mg extract) presence was detected (Table 2).

**Table 2:** HPLC-DAD analysis of Methanol Ethyl Acetate (MEA) Extracts of *Atriplex lasiantha*

Standard	GA	Rut	CA	Cat	Api	Myr	Quer	Kaem
λ (nm)	257	257	325	279	325	368	368	368
μgmg^**− 1**^	ND	0.3	ND	ND	ND	ND	ND	ND

λ (nm) represents wavelength in nanometer while μg mg^−1^ represents the quantity of polyphenol in microgram per milligram of the extract. ND: Not Detected, GA: Gallic Acid, Rut: Rutin, CA: Caffeic Acid, Cat: Catechin, Api: Apigenin, Myr: Myrecetin, Quer: Quercetin, Kaem: Kaempferol

### Antioxidant assays

**Fig. 4:**
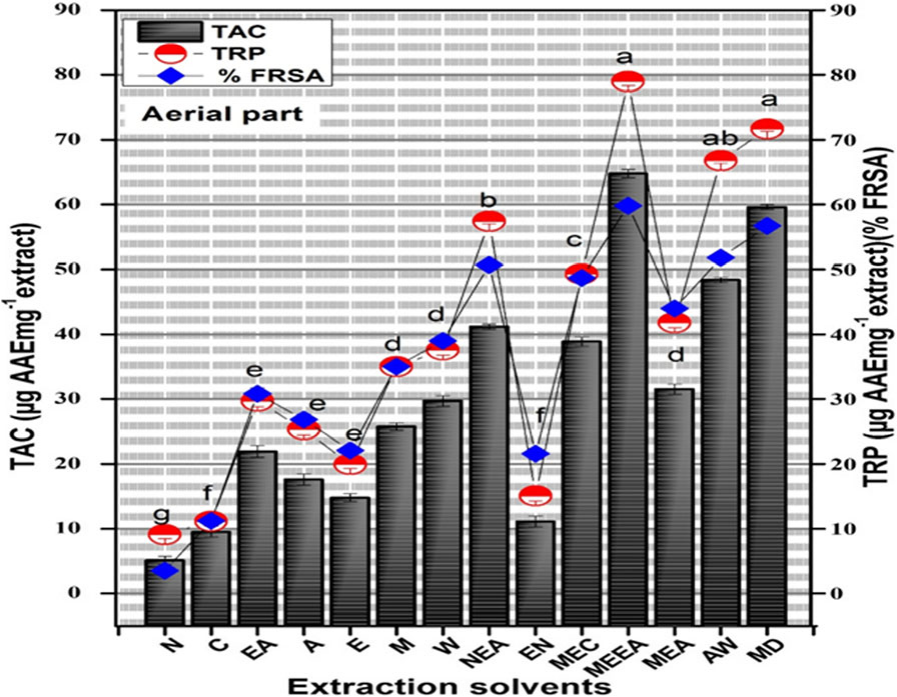
TAC (Total antioxidant capacity, μg AAE/mg plant extract), TRP (Total reducing power, μg AAE/mg plant extract) and FRSA (free radical scavenging activity) determination in aerial part of *A. lasiantha.* Results were presented as mean ± S.D. (*n* = 3). N: n-hexane, C: Chloroform, EA: Ethyl acetate, A: Acetone, E: Ethanol, ME: Methanol, W: Distilled water, NEA: *n*-hexane:ethyl acetate, EN: ethanol:*n*-hexane, MEC: methanol:chloroform MEEA: Methanol-Ethyl acetate, MEA: methanol:acetone, AW: acetone:water, MED: methanol:water

Different mechanisms are involved such as free radical scavenging, prevention of chain initiation and peroxide decomposition. There is no single method which can offer a comprehensive, accurate and quantitative estimate of antioxidant efficiency and antiradical efficacy [41]. Therefore, more than one method is recommended to ratify the presence of antioxidant efficacy of different crude of *A. lasiantha*. To establish the antioxidant nature of the different extracts of *A. lasiantha,* free radical scavenging, Total Antioxidant Capacity and Total Reducing Power assays were carried out as shown in Fig. 4.

#### DPPH free radical scavenging activity

Among all extracts MEEA and MEW showed highest scavenging potential i.e. 59.8 ± 1.07% and 56.7 ± 1.08% respectively at 200 μg/ml (Fig. 4). For other fractions, the scavenging efficiency was in in increasing order of MEEA > MEW > EAN > AW > MEC > MEA > W > ME > EA > A > E > EN > C > N. The free radical scavenging results are dependent on presence of OH group in polyphenols and it is documented that scavenging is the basic contrivance of action of flavonoids [42]. In our study a significant positive relationship exist between TPC and FRSA (R^2^ = 0.9575). Consequently, it can be concluded that different polyphenols in *A. lasiantha* extracts are responsible for antioxidant activity.

#### Total antioxidant capacity by Phosphomolybdenum method

Total antioxidant capability was estimated by phosphomolybdenum method based on formation on phosphate molybdate complex (green in color). The extracts reduced Mo (VI) to Mo (V) and give maximum absorbance at 695 nm. Methanol-ethyl acetate extract exhibited highest antioxidant capability i.e. 64.8 ± 1.16. The antioxidant have a strong correlation with the solvent that was used and a positive linear correlation (R2 = 0.8402) was demonstrated between TPC and antioxidant activity which is in strong agreement with earlier reported documentation of different authors [43].

#### Total reducing power

Reduction potential of *A. lasiantha* was estimated by potassium ferricyanide method based on conversion of ferric chloride to ferrous chloride which results change in color from yellow to green. The intensity of appearance of green color is directly proportion to the sample reducing power [44]. Highest reducing power was observed in methanol-ethyl acetate extract i.e. 79 ± 1.09 μg AAE/mg, while other fractions, the activity was in increasing order of; MEEA > MEW > AW > EAN > MEC > MEA > W > ME > EA > A > E > EN > C > N. These results specify the compatibility with the documentation of previous authors [45] and demonstrate that reduction potential is polarity dependent. Polyphenols of plant act as a good hydrogen and electron donors and capable to terminate chain reaction by conversion of reactive oxygen species and free radicals to more stable product.. In our current investigation, a linear positive correlation was found between TAC, TRP (R2 = 0.9877) and between RSA and TRP (R2 = 0.9258) with a good correlation coefficient that signify the presence of antioxidants.

### Antimicrobial activity

#### Antibacterial activity

The antibacterial potential demonstrated by various samples of *A. lasiantha* reveals that the N, C and EA show almost the same activity against various bacterial strain and all the three samples are moderately active against all the test strains. An average of approximately 7 mm inhibitory zone has been exhibited by N, C and EA against *K. pneumonia, M. luteus* and *B. subtilis,* which is significantly small in comparison with the positive control i.e., Cefixime, which exhibited greater than 20 mm zone of inhibition against majority of the test strains as shown in the Table 3. Curing impact of a plant against infectious diseases has been confirmed by ethnobotanical data where it is reported that 40–80% of antimicrobials have been acquired from medicinal plants [46]*.* Medicinal plants with therapeutic uses can be screened for the presence of antimicrobial entities. As a first step in investigating, the antimicrobial assays provides a glimpse of the microbio-cidal or microbio-static nature. In present study, the susceptibility of extracts was evaluated by agar disc diffusion method. The extracts exhibited varying degree of growth inhibition of the strains under investigation (Table 3). AW extract revealed highest antibacterial activity against *B. subtilis* with 11 ± 0.65 mm zone of inhibition. All the other extracts exhibited moderate activity against *K. pneumoniae, M. luteus* and *E. coli* strains. None of the extracts was active against *S. aureus.*

**Table 3:** Antibacterial activity of *A. lasiantha* extracts against pathogenic bacteria

Extracts Codes	Diameter of growth zone inhibition Zone (mm ± SD at 100 μg/disc; MIC: μg/ml)									
	Gram negative bacteria						Gram positive bacteria			
	*K. pneumoniae*	MIC	*M. luteus*	MIC	*E.coli*	MIC	*B. subtilis*	MIC	*S.aureus*	MIC
N	7.9 ± 0.45	> 100***	7 ± 0.97	> 100***	8.6 ± 1.20	> 100***	7.1 ± 0.62	> 100***	–	–
C	7.7 ± 0.9	> 100***	8 ± 0.43	> 100***	8.3 ± 0.9	> 100***	7.4 ± 1.04	> 100***	–	–
EA	7.2 ± 0.61	> 100***	9 ± 0.57	> 100***	7.5 ± 0.78	> 100***	7 ± 0.36	> 100***	–	–
A	–	–	–	–	7.5 ± 0.54	> 100***	–	–	–	–
E	7.1 ± 0.54	> 100***	8 ± 0.68	> 100***	7.1 ± 0.69	> 100***	–	–	–	–
M	7.0 ± 0.82	> 100***	–	–	8.5 ± 1.2	> 100***	–	–	–	–
W	–	–	–		–	–	–	–	–	–
NEA	8.5 ± 0.29	> 100***	7 ± 0.43	> 100***	7.1 ± 0.25	> 100***	–	–	–	–
EN	7.3 ± 0.84	> 100***	8 ± 0.53	> 100***	7.3 ± 0.19	> 100***	–	–	–	–
MEC	8.1 ± 0.32	> 100***	–	–	7.1 ± 0.23	> 100***	–	–	–	–
MEEA	–	–	–	–	–	–	9 ± 0.74	100***	–	–
MEA	8.3 ± 0.63	> 100***	–	–	–	–	–	–	–	–
AW	–	–	–	–	–	–	11 ± 0.65	100***	–	–
MD	–	–	–	–	–	–	–	–	–	–
PC	12 ± 1.12	1.56	23 ± 1.04	0.78	28 ± 1.09	0.39	21 ± 0.9	3.12	14 ± 0.7	6.25

Results were presented as mean ± S.D. (*n* = 3). ***: *P* < 0.001. -- = No activity, PC: Positive Control (Cefixime), N: *n*-hexane, C: Chloroform, EA: Ethyl acetate, A: Acetone, E: Ethanol, M: Methanol, W: Distilled water, NEA: *n*-hexane-Ethyl acetate, EN: ethanol-*n*-hexane, MEC: methanol-chloroform MEEA: Methanol-Ethyl acetate, MEA: methanol-acetone, AW: acetone-water, MD: methanol-water

#### Antifungal activity

The antifungal activity of various solvent samples of *A. lasiantha* has been summarized in Table 4. The statistical analysis shows that the results of all the test samples are significantly different from those of the values of positive control. The zone of inhibition demonstrated by all the solvent extracts reveal a moderate antifungal potential which can also be perceived from the MIC values. Emergence of microbial drugs resistance is due to its genetic ability to acquire and transmit resistance against the drugs which are consumed as therapeutic mediators [47]. In present study, the susceptibility of extracts was evaluated against filamentous fungi by agar disc diffusion method. The extracts which shows zone of inhibition ≥10 mm were evaluated for their minimum inhibitory concentration through micro dilution method. The extracts demonstrated significant zones of growth inhibition against test strains (Table 4). Among the aerial part extracts of *A. lasiantha*; NEA and EA extracts exhibited highest activity against *A. fumigatus* with 14 ± 1.94 and 12 ± 0.31 mm growth inhibition zone respectively. *F. solani* strain was also sensitive against NEA extract with 10 ± 0.76 mm zone of growth inhibition. On the other hand, all the extracts demonstrated moderate activity against *A. flavus*, *A. niger* and *Muco*r spp. respectively.

**Table 4:** Antifungal activity of *A. lasiantha* against various fungal strains

Extracts/ Samples	Diameter of growth inhibition zone (mm)									
	*F. solani*	MIC μg/ml	*A. fumigatus*	MIC μg/ml	Mucor spp.	MIC μg/ml	*A. flavus*	MIC μg/ml	*A. niger*	MIC μg/ml
N	–	–	–	–	7 ± 0.43	100***	7 ± 0.44	100***	7 ± 0.39	100***
C	–	–	8 ± 0.51	100***	–	–	–	–	–	–
EA	–	–	12 ± 0.31	100***	–	–	–	–	7 ± 0.25	100***
A	–	–	–	–	–	–	7 ± 0.29	100***	–	–
E	8 ± 0.46	100***	9 ± 0.67	100***	–	–	–	–	–	–
M	8 ± 0.52	100***	–	–	7 ± 0.39	100***	–	–	–	–
W	–	–	10 ± 1.09	100***	–	–	–	–	7 ± 0.37	100***
NEA	10 ± 0.76	100***	14 ± 1.94	50***	8 ± 0.46	100***	–	–	–	–
EN	–	–	–	–	–	–	8 ± 0.53	100***	8 ± 0.53	100***
MEC	–	–		–	–	–	–	–	–	–
MEEA	8 ± 0.49	100***	–	–	7 ± 0.87	100***	–	–	–	–
MEA	–	–	7 ± 0.47	100***	–	–	7 ± 0.37	100***	–	–
AW	–	–	–	–	–	–	–	–	–	–
MD	–	–	8 ± 0.81	100***	–	–	9 ± 0.71	100***	7 ± 0.42	100***
PC	30 ± 1.54	1.56	32 ± 1.15	3.12	28 ± 0.87	6.25	30 ± 1.46	1.56	34 ± 2.04	0.78

Results were presented as mean ± S.D., *n* = 3, ***: *P* < 0.001. -- = No activity; N: *n*-hexane, C: Chloroform, EA: Ethyl acetate, A: Acetone, E: Ethanol, ME: Methanol, W: Distilled water, NEA: *n*-hexane:ethyl acetate, EN: ethanol: *n*-hexane, MEC: methanol:chloroform MEEA: Methanol-Ethyl acetate, MEA: methanol:acetone, AW: acetone: water, MED: methanol:water. PC: Positive control (Clotrimazole)

#### Alpha amylase inhibition activity

**Fig. 5:**
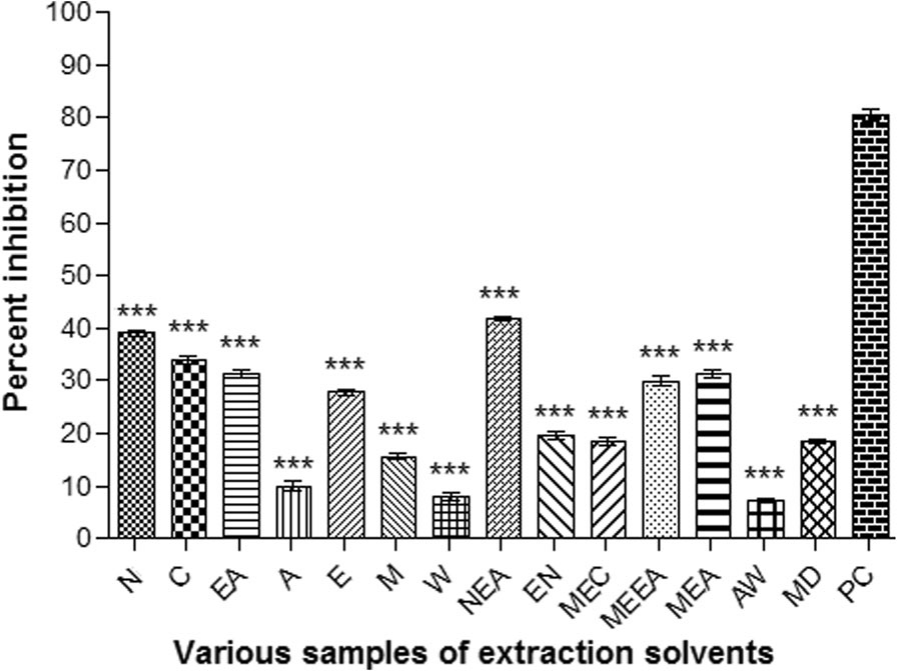
Percent α-amylase inhibitory potential of various samples of extraction solvents. Results were presented as mean ± S.D.; n = 3, ***: *P* < 0.001. Nh: *n*-hexane, C: Chloroform, EA: Ethyl acetate, A: Acetone, E: Ethanol, M: Methanol, W: Distilled water, NEA: *n*-hexane-Ethyl acetate, EN: ethanol-*n*-hexane, MEC: methanol-chloroform MEEA: Methanol-Ethyl acetate, MEA: methanol-acetone, AW: acetone-water, MD: methanol-water, PC: Positive control (Acarbose)

The effect of *A. lasiantha* against α-amylase has been summarized in Fig. 5. In our present study, highest percentage of α-amylase inhibition was observed in EthNh extract of *A. lasiantha* aerial part i.e. 41.8 ± 1.09% and inhibition was decreased in the order of EAN > N > C > MEA = EA > MEEA > E > EN > MEC > MEW> ME > A > W >. AW. Medicinal plants have been attaining substantial attention due to numeral active constituents which act as hypoglycemic mediators [48]. As far as the positive control is concerned, the Acarbose has been employed against this enzyme which exhibited significant inhibitory activity against the alpha amylase but in comparison with the test sample we can deduce that the anti-alpha amylase potential of various solvent samples of A. lasiantha is significantly smaller, which is obvious from the statistical analysis.

#### Protein kinase inhibition potential

In the current study, *Streptomyces* 85E strain was utilized to evaluate the kinase inhibitory potential of the given extracts of *A. lasiantha*. Using *Streptomyces* as a recommended strain for kinase inhibition assay attribute to the identification of a wide range of eukaryotic kinase modulators, probably because the *Streptomyces* enzymes are evolutionary forerunners of their highly specific eukaryotic counterparts. Among aerial part extracts of *A. lasiantha*, MEA extract demonstrated significant inhibition of hyphae formation at 100 μg/disc with maximum activity with 11 ± 0.49 mm bald zone of inhibition (Table 5).

**Table 5:** HepG2 Cytotoxicity, Streptomyces hyphae formation inhibition and Anti-leishmanial capability of various solvent extracts

Extract/ Samples	Hep G2 Cytotoxicity		Protein kinase inhibition		Antileishmanial capability	
	inhibition%	IC_50_ (μg/ml)	Diameter of zone of inhibition (mm)		Mortality %	LC_50_ μg/ml
			Clear zone	Bald zone		
N	–	–	–	7 ± 0.37***	71 ± 0.49	12.8***
C	10 ± 1.5	204***	–	8 ± 0.81***	–	–
EA	18 ± 1.01	101***	–	9 ± 0.49***	–	–
A	3 ± 0.9	773***	–	7 ± 0.97***	–	–
E	7 ± 0.89	312***	–	8 ± 0.43***	–	–
M	21 ± 0.37	096***	–	9 ± 0.57***	–	–
W	–	–	–	–	71 ± 0.93	16.8***
NEA	–	–	–	8 ± 0.68***	72 ± 0.76	11.5**
EN	16 ± 1.34	168***	8 ± 0.34	–	–	–
MEC	39 ± 1.01	046***	9 ± 0.41	–	–	–
MEEA	–	–	8 ± 0.70	–	–	–
MEA	12 ± 0.57	191***	–	11 ± 0.49***	–	–
AW	15 ± 0.83	149***	9 ± 0.51	–	–	–
MD	–	–	8 ± 0.68	–	–	–
PC	57 ± 2.56	5.10	–	21 ± 1.02	79 ± 1.15	0.01

Results were presented as mean ± SD; n = 3; The percent potential of each sample has been expressed at 100 μg/mL **: *P* < 0.01, ***: *P* < 0.001. -- = Not active, N: *n*-hexane, C: Chloroform, EA: Ethyl acetate, A: Acetone, E: Ethanol, M: Methanol, W: Distilled water, NEA: *n*-hexane-Ethyl acetate, EN: ethanol-*n*-hexane, MEC: methanol-chloroform MEEA: Methanol-Ethyl acetate, MEA: methanol-acetone, AW: acetone-water, MD: methanol-water, PC: Positive control; Doxorubicin employed in cytotoxicity assay, Surfactin in Protein kinase inhibition assay, Amphotericin-B in Antileshmanial assay

#### Cytotoxicity against Hep G2 cell line

Results of all samples tested at 100 μg/mL (20 μL) is summarized in Table 5. Among all extracts, MEC extract showed high antiproliferative activity with an inhibition of 39 ± 1.01%. It has been reported that numerous exogenous antioxidants act as a mediator for the free radicals that might be linked with the proliferation of cancer. The difference among the potential of various samples against various targets may be attributed to the presence of different types of secondary metabolites in the solvent extract, which may differ on the base of their polarity.

#### Antileishmanial activity

In present study, antileishmanial potential of *A. lasiantha* was first time evaluated against *Leishmania tropica*. In case of aerial part of *A. lasiantha*, NEA, N and W extracts showed significant leishmanicidal potential with 50% mortality at 13.5 μg/mL, 12.8 μg/mL and 16.8 μg/mL respectively (Table 5). Maximum mortality rate may be attributed to the existence of those compounds which are detected by HPLC like rutin which have been reported effective against antitrypanosomal and antileishmanial agent or might be due to any other polyphenol which are not detected by HPLC.

### Hemolytic activity

**Fig. 6:**
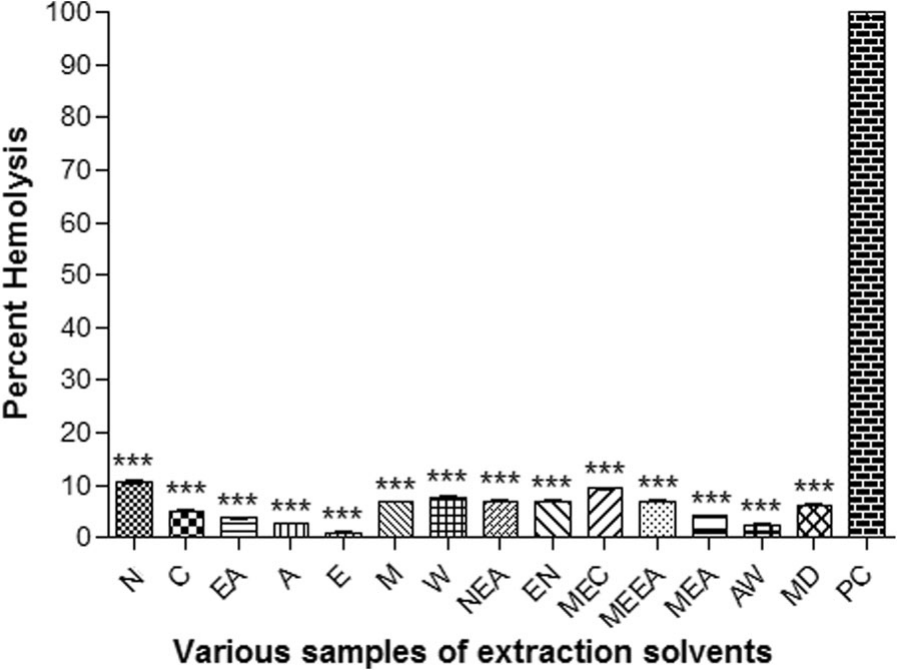
Percent hemolytic potential of various samples of extraction solvents. Results were presented as mean ± S.D.; n = 3; ***: P < 0.001. N: *n*-hexane, C: Chloroform, EA: Ethyl acetate, A: Acetone, E: Ethanol, M: Methanol, W: Distilled water, NEA: *n*-hexane-Ethyl acetate, EN: ethanol-*n*-hexane, MEC: methanol-chloroform MEEA: Methanol-Ethyl acetate, MEA: methanol-acetone, AW: acetone-water, MD: methanol-water, PC: Positive control (Triton-X100)

Erythrocytes are extensively used as a prototype to direct the toxicity of drug as well as to direct the general indication of membrane toxicity. There is another advantage that blood is easily available and isolation is quite easy; furthermore, its membrane structure is similar to cell membrane structure of other cells [49]. In our current study, only chloroform extract indicated about 10% hemolysis, while other test samples indicated hemolysis < 10% concluding their compatibility (Fig. 6).

## Discussion

Results of the current investigational studies show that diverse variety of chemical components, their accessibility for extractable solvents is the rudimentary optimal feature that influence the extraction yield. Extraction efficiency as well as biological activities are highly dependent on nature of solvent system. Moreover, it has been suggested that maceration along with amalgamation of different solvents might be a better option for extraction of plant secondary metabolites [50]. Among the secondary metabolites, the phenolics and flavonoids have considerable physiological and biochemical significance in plants. These bioactive compounds exhibit diverse biochemical functions and protection against various pathological disturbances or oxidative degradation by scavenging free radicals [51]. In addition, flavonoids are important antioxidants by acting as hydrogen donors, singlet oxygen quenchers and reducing agents. They also have metal chelation property owing to the presence of hydroxyl group. This chelation could be critical in preventing the generation of biomolecule targeting radicals [52]. It is well documented that naturally occurring phenolics and flavonoids display antioxidant activities, scavenge free radicals and preclude the mutilation triggered by them. Significant Positive correlation exists between phenolics and flavonoid content in various plants, which reveals that antioxidant potential of phenols might be attributed to presence of flavonoids. In the same way, the activity demonstrated by the plant extracts in the current study might also be attributed to the synergistic or antagonistic belongings of phytochemicals of plant [53].

Moreover as far as the use of alpha amylase is concerned, it is involved in the breakdown of complex carbohydrates into smallest glucose units in the gastrointestinal tract. The resultant glucose units are absorbed into the systemic circulation, which leads to increase in blood sugar level. So the inhibition of enzyme amylase leads to inhibition of breakdown of carbohydrates and so there is decreased entry of glucose into the systemic circulation. The alpha amylase inhibitors have a prominent role in antidiabetic therapy and one of the biological potential evaluated in the current study was based on antidiabetic activity of plant extract which were figured out via alpha amylase inhibitory potential. Likewise, protein kinase (PK) inhibition is a popular strategy in the treatment of cancer. Plants have proved as a vital resource of anticancer compounds. Most of inhibitors are investigated for cancer but deregulation of kinase function is also involved in neurological, immunological, infectious and metabolic diseases. Researchers are diverted for development of kinase inhibitors, so cancer along with these disorders can be treated [47]. There are different methods for the measurement of inhibition of kinases. PK enzyme is accountable for phosphorylation of tyrosine and serine/threonine amino residues which play a key role in cell proliferation, cell differentiation, metabolism and apoptosis. Uncontrolled phosphorylation at serine/threonine and tyrosine amino acid residues of protein kinase leads to the onset of cancer. There are different methods for the measurement of inhibition of kinases. Protein kinase inhibitors present in samples block the aerial hyphae formation of *Streptomyces sp,* induced by kinases, thus may be hypothesized to inhibit the cancer cell proliferation [54]. A great number of kinases especially receptor protein tyrosine kinases (RPTKs) are known to be of considerable importance in the arena of oncology. Protein phosphorylation, which regulates various aspects like cell division, survival and apoptosis, involves the transfer of phosphate group from ATP to several acceptor residues like serine, threonine and tyrosine kinase [55]. Signal transduction occurs in eukaryotes via eukaryotic protein kinases (ePK) which phosphorylate either serine-threonine (ser/ther specific protein kinase; STPKs), tyrosine (tyrosine specific protein kinases; TPKs), or both threonine and tyrosine [56, 57]. Unusual activity of protein kinases either as overexpression, mutation or deregulation evades the physiological processes [58] and leads to the development of pathological processes such as malignancy and viral infections. *Streptomyces* are of considerable importance owing to their ability to produce variety of antibiotics. Their multicellular differentiation and complex life cycle require kinases for various levels of regulation and signal transduction mechanisms [54]. Genome sequencing projects have assisted researchers in identification of various eukaryotic protein kinases in mycobacterium, cyanobacteria and *Streptomyces* [59]. AfsR, PKaB, PkaA, PKg2, PKg3 and PKg4 are the prominent examples of kinases detected so far while PKg2 is the one needed in hyphae formation [60]. In the current study different extracts exhibited significant kinase inhibition potential and can be a source of potent chemopreventive agents.

One of the biological assays in the current investigational studies was against Leishmaniasis. Leishmaniasis has been reported to cause the ninth largest disease burden among infectious diseases [61]. The disease is caused by kinetoplastid of the genus *Leishmania* which is transmitted by the female sand fly. The statistics show that according to the geographical distribution of the sand fly, around 350 million people are at risk of infection worldwide. Around 14 million people in Asia, Africa, Europe and the Americas are prone to be affected directly by this disease. It has been calculated that around 70,000 deaths a year is caused by Leishmaniasis hence putting it among the top ranking communicable diseases [62, 63].

Most importantly, many of the antimicrobial compounds also manifest cytotoxicity in the eukaryotic cells, which limits their applications for therapeutic uses. This also necessitates the prior screening of test plant materials for hemolytic activities, which has also been carried out the current investigational studies with acceptable ranges.

## Conclusion

Plant materials are a robust source of potential bioactive compounds/phytochemicals with diverse physicochemical characteristics. Due to chemical diversity and natural affability of natural products, either in the form of standardized extract or as a pure compound based on ethnopharmacological properties plays a s significant role in new drug development. The most commonly employed one or two extraction solvents do not extract all the potentially bioactive constituents due to their varied polarity. The bioactive extracts of *A. lasiantha* were optimized using fourteen solvents systems based on their polarity to narrow down the search and isolation of bioactive compounds. Significant cytotoxic and kinases inhibiting potentials of extracts suggests bioguided isolation of anticancer compounds and further detailed studies. Antioxidant and anti-leishmanial results also suggests that the plant might be a source of phytoconstituents responsible for these activities and thus warrant further studies for isolation of compounds effective in free radicals induced degenerative disorders and Leishmaniasis.

## Abbreviations

A: Acetone
ATCC: American Type Culture Collection
AW: Acetone: water
C: Chloroform
CFU: Colony Forming Units
DPPH: 2,2-diphenyl-1-picryhydrazyl
E: Ethanol
EA: Ethyl acetate
EN: ethanol: n- hexane
ePKs: eukaryotic protein kinases
FCBP: First *Fungal* Culture Bank of Pakistan
FCR: Folin-Ciocalteu reagents
HepG2: Hepatoma G_2_ Cells
HPLC-DAD: High-performance liquid chromatography diode-array detection system
KP: Khyber Pakhtunkhwa
ME: Methanol
MEA: methanol: acetone
MEC: methanol: chloroform
MED: methanol: water
MEEA: Methanol-Ethyl acetate
MIC: Minimum inhibitory concentration
N: *n*-hexane
NEA: *n*-hexane ethyl acetate
PBS: Phosphate Buffer Saline
PKs: Protein kinase
ROS: Reactive Oxygen Species
RPTKs: Receptor protein tyrosine kinases
SDA: Sabouraud dextrose agar
STPKs: Ser/ther specific protein kinase
TFC: Total Flavonoid Content
TPC: Total Phenolic Content
TPKs: Tyrosine (tyrosine specific protein kinases
TSB: Ttryptone Soy Broth
W: Distilled water
WHO: World Health Organization
μg AAE/mg extract: Ascorbic Acid Equivalent per microgram extract
